# Associations of adverse childhood experiences with blood pressure among early adolescents in the United States

**DOI:** 10.1016/j.ajpc.2024.100883

**Published:** 2024-10-04

**Authors:** Abubakr A.A. Al-shoaibi, Christopher M. Lee, Julia H. Raney, Kyle T. Ganson, Alexander Testa, Erin E. Dooley, Holly C. Gooding, Kelley Pettee Gabriel, Fiona C. Baker, Jason M. Nagata

**Affiliations:** a Department of Pediatrics, Division of Adolescent and Young Adult Medicine, University of California San Francisco, San Francisco, CA, USA; b Factor-Inwentash Faculty of Social Work, University of Toronto, Toronto, ON, Canada; c Department of Management, Policy and Community Health, University of Texas Health Science Center at Houston, Houston, TX, USA; d Department of Epidemiology, University of Alabama at Birmingham, Birmingham, AL, USA; e Division of General Pediatrics and Adolescent Medicine, Department of Pediatrics, Emory University School of Medicine, Atlanta, GA, USA; f Center for Health Sciences, SRI International, Menlo Park, CA, USA; g School of Physiology, University of the Witwatersrand, Johannesburg, South Africa

**Keywords:** Adolescents, Adverse childhood experiences, Systolic blood pressure, Diastolic blood pressure

## Abstract

The associations of adverse childhood experiences (ACEs) with blood pressure in adulthood are inconclusive. Similarly, the association between ACEs and blood pressure earlier in the life course is understudied. This study aims to assess the associations of ACEs with blood pressure among early adolescents. We utilized data collected at baseline (age: 9–10 years) and Year 2 follow-up from 4077 participants in the Adolescent Brain Cognitive Development (ABCD) Study. We used adjusted multiple linear regression models to estimate the associations of ACEs (cumulative score and subtypes) at baseline with systolic blood pressure (SBP) and diastolic blood pressure (DBP) at year 2 of follow-up. Experiencing ≥4 ACEs (compared to 0) was significantly associated with higher SBP (*B* = 3.31, 95 % CI 0.03, 6.57, *p* = 0.048). Of the ACEs subtypes, household substance use (*B* = 2.28, 95 % CI 0.28, 4.28, *p* = 0.028) and divorce or separation (*B* = 2.08, 95 % CI 0.01, 4.15, *p* = 0.048) were both significantly associated with a higher SBP while household mental illness (*B* = 2.57, 95 % CI 1.32, 3.81, *p* < 0.001) was significantly associated with a higher DBP. Our findings suggest that exposure to multiple ACEs is associated with higher blood pressure in adolescence.

## Graphical abstract

**Figure:**
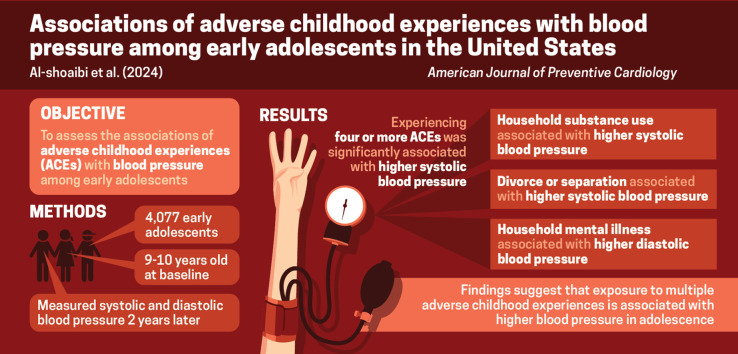

## 1 Introduction

Adverse childhood experiences (ACEs), including sexual, physical, and emotional abuse, violence, growing up in a family with mental illness, and substance use that occurs before the age of 18, can have an impact on health and well-being that may last until adulthood [1]. Approximately 64 % of adults in the United States (U.S.) have experienced at least one type of ACE; over 17 % have reported experiencing four or more types [2]. ACEs in childhood are associated with subsequent high-risk behaviors such as smoking, alcohol initiation [3] and physical inactivity [4] along with poor mental and physical health outcomes such as depression [5], diabetes [6], and cancer [7].

According to the Centers for Disease Control and Prevention (CDC), 1 in 25 youth ages 12–19 years (an estimated 1.3 million individuals) have hypertension (defined as systolic blood pressure (SBP) and/or diastolic blood pressure (DBP) ≥95th percentile for children aged <13 years old or ≥130/80 mm Hg, and ≥130/80 mm Hg for children aged ≥13 years old), and 1 in 10 have elevated blood pressure (defined as SBP and DBP ≥90th percentile to <95th percentile or ≥120/80 mm Hg to <95th percentile for children aged <13 years old, and 120/<80 to 129/<80 mm Hg for children aged ≥13 years old) [8,9]. Higher blood pressure in childhood is associated with a greater risk for cardiovascular disease (CVD) in adulthood, highlighting the importance of blood pressure control in early life [10]. Because blood pressure is a modifiable risk factor, understanding the factors that influence blood pressure in childhood is critical to preventing CVD in adulthood.

Whether ACEs are associated with high blood pressure in adulthood is inconclusive [[11], [12], [13]]. A systematic review examining the link between ACEs and heightened blood pressure among women in the U.S. reported a significant positive association between exposure to ACEs and blood pressure in 6 out of the 12 studies meeting inclusion criteria [13]. Using data from the National Longitudinal Study of Adolescent Health, another study looking at the relationship between childhood maltreatment and hypertension in young adulthood found an association between sexual abuse in early childhood and hypertension in women but not men [11]. Finally, the Georgia Stress and Heart Study, a 23-year longitudinal study, found that individuals exposed to multiple ACEs demonstrated a more pronounced rise in blood pressure during young adulthood in comparison to those without ACEs [12]. This inconsistency in results from the aforementioned studies could be attributed to differences in blood pressure measurement (measured by the individual vs. clinical measurement), types of ACEs assessed, and population characteristics [13]. Potential mechanisms linking ACEs with high blood pressure could be stress-related hormonal imbalances and unhealthy lifestyle habits, both of which may impact blood pressure regulation [14].

Even less is known about the potential relationship between ACEs and blood pressure in children and adolescents. To our knowledge, there have been only two studies that have assessed the association between ACEs and blood pressure in children and youth [15,16]. While one study did not find an association between ACEs and hypertension [16], the other found that greater than or equal to 4 ACEs was associated with higher DBP [15]. Both studies were cross-sectional and consisted of relatively small sample sizes. Identifying the associations of ACEs (and ACE subtypes) and blood pressure during the transition from childhood to adolescence can inform strategies for the prevention and management of high blood pressure. Therefore, we used data from the Adolescent Brain Cognitive Development (ABCD) study to determine if ACEs are prospectively associated with higher blood pressure in early adolescents. We hypothesized that ACEs in early life would be associated with high blood pressure two years later.

## 2 Materials and methods

The ABCD study, a large-scale longitudinal study of adolescent cognitive and physical health [17], recruited 11,875 youth aged between 9 and 10 years old at baseline (2016–2018) by employing epidemiological sampling methods to accurately represent the diversity of the U.S. adolescent population [17]. Baseline and year 2 of follow-up data were analyzed. Participants with missing data on blood pressure measures at year 2 of follow-up (*n* = 7534) and covariates at baseline (*n* = 364) were excluded, leaving 4077 individuals for the current analysis (see Supplementary Table 1 for a comparison of characteristics of included and excluded participants). Approval for the study was obtained from the centralized Institutionalized Review Board (IRB) at the University of California at San Diego and individual study site IRBs. Participant caregivers gave written, informed consent, and participants provided their written assent [17].

The ACEs score was calculated based on the responses of adolescents and parents to the baseline survey (2016–2018). The ABCD study assesses nine out of ten ACEs which correspond to the items in the original CDC-Kaiser surveys [18], including physical abuse, sexual abuse, emotional neglect, physical neglect, household substance use, household divorce or separation, household mental illness, household violence, and household criminal justice involvement (see Appendix A for details about each ACE). The ACEs cumulative score was computed by summing up the “yes” responses from either the child or caregiver responses for any of the nine subtypes of ACEs at baseline based on prior research, with “yes” responses indicating endorsement of ever experiencing an event (e.g., physical abuse, sexual abuse) in the participant's lifetime [19]. The ACEs score was then categorized into five groups: 0, 1, 2, 3, and 4+, following the findings from prior research that suggest four or more ACEs were associated with a higher risk of negative physical and mental health outcomes (details about the questions that adolescents/caregivers were asked are shown in Appendix A) [4,[18], [19], [20], [21]].

We used the blood pressure measurement that was recorded on a single day at Year 2 follow-up of the ABCD study [16]. Trained research assistants measured blood pressure using an automatic sphygmomanometer (OMRON HEM 907 XL, MicroLife USA, Inc, Dunedin, FL). Blood pressure was measured using the right arm unless it was not feasible. The participants rested for 5 minutes in a quiet environment before blood pressure was taken seated with arm and back support, legs uncrossed, and feet flat on the floor. The right arm was rested palm-up on a table. To ensure proper cuff size, the circumference of the mid-upper arm was measured before selecting one of the available cuff sizes. The average blood pressure was calculated from three measurements (in millimeters of mercury [mmHg]) separated by 60 second rest intervals. Systolic blood pressure (SBP) and diastolic blood pressure (DBP) were converted into age- and sex-specific percentiles, from which hypertensive blood pressure readings were determined based on the American Academy of Pediatrics guidelines for age and sex [9]. Hypertension was categorized into two groups: normal (SBP and DBP) defined as <95th percentile for children aged <13 years old or SBP and DBP <130/80 mm Hg for children aged ≥13 years old) and hypertensive defined as (SBP and/or DBP) ≥95th percentile for children aged <13 years old or ≥130/80 mm Hg, and ≥130/80 mm Hg for children aged ≥13 years old).

The following covariates were included in this study: race/ethnicity (Asian, Black, Latino/Hispanic, Native American, Other), sex (female, male), household income (less than $75,000 vs. more than $75,000), parent education (high school or less vs. college or more), and participant age (years). Body mass index (BMI) (kg/m^2^) was measured and converted into a percentile. Data on race/ethnicity, sex, income, and education were collected from the parent self-report at baseline. Geographic variation was also adjusted for as a confounder using study site location.

Multivariable linear and logistic regression models were used to assess the associations of 1) cumulative ACEs scores and 2) ACEs subtypes with SBP, DBP, and hypertension. Models were adjusted for age, sex, race/ethnicity, household income, parent education, data collection site, and BMI. We also assessed the associations of cumulative ACEs score and its subtypes with SBP and DBP, adjusting for the same covariates with the exception of parent education at years 1 and 2, and household income at years 1 and 2. Furthermore, we tested the correlation and multicollinearity between ACEs, household income, and parent education. Propensity weighting based on the American Community Survey provided by the U.S. Census was applied to the analysis to provide representative population estimates [22]. All the analyses were performed using STATA 18.0 software. Two-sided *P* < 0.05 was used to indicate statistical significance.

## 3 Results

The analytic sample mean age at baseline was 9.9 ± 0.6 years, 49.4 % of participants were female, 45.0 % were from a racial or ethnic minority, 55.3 % reported annual household incomes of less than $75,000, and 82.8 % of participants had caregivers who had received a college education or more (Table 1). Nearly 80 % of youth reported experiencing at least 1 ACE in their lifetime.

**Table 1:** Sociodemographic characteristics, adverse childhood experiences (ACEs), and blood pressure percentiles of Adolescent Brain Cognitive Development (ABCD) Study participants (Year 2, *N* = 4077).

Sociodemographic characteristics (baseline)	Mean (SD) /%
Age (years)	9.9 (0.6)
Sex, n (%)	
Female	49.4 %
Male	50.6 %
Race/ethnicity (%)	
White	55.0 %
Latino / Hispanic	16.6 %
Black	16.6 %
Asian	5.7 %
Native American	3.8 %
Other	2.2 %
Household income (%)	
Less than $75,000	55.3 %
≥$75,000	44.7 %
Parents with college education or more (%)	82.8 %
Number of ACEs	
0	20.3 %
1	29.3 %
2	21.4 %
3	12.7 %
4+	16.4 %
ACEs type	
Physical abuse (%)	0.9 %
Sexual abuse (%)	0.8 %
Emotional neglect (%)	1.2 %
Physical neglect (%)	53.0 %
Household substance use (%)	43.8 %
Household divorce or separation (%)	15.3 %
Household mental illness (%)	33.2 %
Household violence (%)	44.5 %
Household criminal justice involvement (%)	14.8 %
Blood pressure measures	
Systolic blood pressure percentile (at Year 2 follow-up), mean (SD)	42.0 (29.2)
Diastolic blood pressure percentile (at Year 2 follow-up), mean (SD)	44.2 (24.4)
Blood pressure categories	
Normal blood pressure (*n* = 3675)	90.3 %
Elevated blood pressure (*n* = 162)	4.0%
Hypertension (*n* = 223)	5.7 %
BMI percentile, mean (SD)	61.8(30.6)

Abbreviations: ACEs, adverse childhood experiences; BMI, body mass index.

Propensity weights were applied to yield estimates based on the American Community Survey from the US Census. SD = standard deviation.

The results of the adjusted linear regression models showed that those who experienced ≥ 4 ACEs, compared to those who experienced 0 ACEs, had a higher SBP percentile (*B* = 3.31, 95 % CI 0.03, 6.57, *p* = 0.048). There was no difference in SBP among those with 1, 2, or 3 ACEs compared to participants without ACEs. No association was observed for DBP (Table 2). Of the ACEs subtypes, individuals who experienced household substance use (*B* = 2.28, 95 % CI 0.28, 4.28, *p* = 0.028) and household divorce or separation (*B* = 2.08, 95 % CI 0.01, 4.15, *p* = 0.048) had a higher SBP percentile compared to those who did not experience these ACEs. Household mental illness was associated with a higher DBP percentile (*B* = 2.57, 95 % CI 1.32, 3.81, *p* < 0.001). The results of adjusted logistic regression models showed that cumulative ACEs scores and ACE subtypes were not significantly associated with blood pressures that met the threshold for hypertension (Supplementary Table 2). The results of models adjusted for covariates from Years 1 and 2 showed that household substance use (*B* = 2.40, 95 % CI 0.40, 4.40, *p* = 0.021) and household mental illness (*B* = 2.52, 95 % CI 1.26, 3.77, *p* < 0.001) remained significant (Supplementary Table 3).

**Table 2:** Associations between baseline adverse childhood experiences and blood pressure percentile at two-year follow-up adjusted for baseline confounders in 4077 participants in the Adolescent Brain Cognitive Development Study.

	Systolic blood pressure		Diastolic blood pressure	
	Adjusted^a^		Adjusted^a^	
	B (95 % CI)	p	B (95 % CI)	p
Number of ACEs				
0	Ref		Ref	
1	−0.49 (−4.08, 3.09)	0.779	0.74 (−1.57, 3.04)	0.514
2	1.32 (−1.41, 4.04)	0.327	1.39 (−0.69, 3.49)	0.180
3	0.45 (−2.76, 3.66)	0.773	1.45 (−1.06, 3.95)	0.243
4+	**3.31 (0.03, 6.57)**	**0.048**	2.37 (−0.52, 5.27)	0.103
ACE subtypes^b^				
Physical abuse	−2.03 (−8.23, 4.17)	0.504	−2.80 (−15.95, 10.34)	0.662
Sexual abuse	7.51 (−2.17, 17.20)	0.122	1.92 (−3.16, 6.99)	0.441
Emotional neglect	−6.00 (−13.31, 1.30)	0.102	−1.89 (−9.72, 5.92)	0.620
Physical neglect	0.61 (−4.40, 5.62)	0.802	3.08 (−0.70, 6.88)	0.106
Household substance use	**2.28 (0.28, 4.28)**	**0.028**	1.35 (−0.49, 3.19)	0.142
Household divorce or separation	**2.08 (0.01, 4.15)**	**0.048**	1.18 (−1.21, 3.58)	0.315
Household mental illness	1.57 (−0.01, 3.16)	0.052	**2.57 (1.32, 3.81)**	**<0.001**
Household violence	−0.71 (−2.93, 1.49)	0.508	−0.34 (−2.08, 1.40)	0.686
Household criminal justice involvement	−0.51 (−3.46, 2.44)	0.723	−2.51 (−6.03, 1.01)	0.152

Abbreviations: ACEs, adverse childhood experiences.

Bold indicates *p* < 0.05.

a Covariates: age, race/ethnicity, sex, household income, parent education, study site, and BMI at baseline.

b Outputs represent the abbreviated output for a series of linear regression models with each ACE subtype as the independent variable and systolic and diastolic blood pressure as the dependent variables. Thus, the table represents the output from nine regression models in total.

## 4 Discussion

In this analysis of a demographically diverse sample of U.S. adolescents, we identified several significant associations between ACEs and blood pressure. First, we found a positive association between 4+ ACEs and higher SBP percentile. Additionally, we identified significant associations of specific ACE subtypes, notably household substance use and household divorce or separation with higher SBP percentile, and household mental illness with higher DBP percentile.

Both behavioral and physiological pathways have been proposed as mechanisms underlying the association between ACEs and blood pressure. One potential mechanism may involve the psychosocial impact of ACEs on the stress response [23]. It has been proposed that exposure to ACEs may disrupt brain development, altering an individual's ability to cope with stress [24,25]. In adults, ACEs have been reported to increase levels of perceived stress and risk of depression [23], which itself has been shown to be a risk factor for hypertension [26].

Physiologic mechanisms center around the idea that many organ systems, including nervous, endocrine, and immune, may be shaped via stress-dependent pathways, which are influenced by ACEs [27]. In particular, hyperactivity of the sympathetic nervous system, a disrupted hypothalamic-pituitary axis, and elevated levels of inflammation have been associated with ACEs [[28], [29], [30]], and these factors in turn have been associated with elevated blood pressure [[31], [32], [33]]. In particular, it has been shown that ACEs are associated with dysregulation of cortisol, the stress hormone [34]. Vascular changes in response to ACEs, including an elevated aortic augmentation index in adolescence (a risk factor for future CVD) [35], elevated plasma ET-1 level (a potent vasoconstrictor) in adolescents [36], and reduced activation of regions of the brain involved in the cardiovascular response to stress [37] may be additional contributing physiologic mechanisms to high blood pressure. These behavioral and physiological mechanisms likely play integral roles in a multifaceted pathway that explains our finding that experiencing 4+ ACEs is associated with a higher systolic blood pressure percentile in adolescence.

The associations observed between household substance use and household divorce or separation with elevated SBP percentile, and between household mental illness and higher DBP percentile in adolescence, may stem from the stressors imposed on children residing in such environments. A prior longitudinal study reported an association between parental separation during childhood and offspring hypertension at age 46 [38]. Parental separation is a stressful process, which may result in a reduction of resources and social connections, and increase the need for psychological assistance [39]. If experienced during the formative years of development, these stressors can have lasting psychosocial (increased rates of anxiety and depression) and biological consequences, such as heightened blood pressure [40,41]. The findings of our study suggest that the effects of ACEs on blood pressure may manifest as early as adolescence. Regarding substance use, it is possible that parental substance use negatively impacts the emotional and behavioral patterns of the whole family [42]. As a result, parental substance use may serve as a stressor for adolescents, a potential explanation for our finding that household substance use correlates with elevated SBP percentile in adolescence.

It is perhaps surprising that our study did not find associations between certain ACE subtypes, such as household violence and sexual abuse, and high blood pressure. In particular, sexual abuse has been consistently shown to be a risk factor for CVD in adult women [43]. It is possible that these associations are more likely to manifest later in life and/or depend on chronicity of exposure. It is also possible that the accuracy of certain questions from the ABCD study to assess ACE exposure was limited. Furthermore, the small sample sizes for some exposures (i.e., sexual abuse) may have limited our analysis.

Strengths of our study include the sociodemographically diverse and large population-based sample, longitudinal study design that allowed for follow-up over a two-year period, and use of objective data via in-office measurements to report blood pressure. However, our study has limitations, including the lack of a validated screener for ACEs, which could have introduced the potential for misclassification, as well as a lack of incorporation of the timing of ACEs experienced by participants into the analysis of data, which may be relevant to their effects on blood pressure [44,45]. The amount of missing data was relatively large, which may have led to a lack of statistical power to find an association between ACEs and hypertension or elevated blood pressure. Additionally, there were notable differences between the individuals included in the analyses and those who were excluded. Specifically, those included had a higher socioeconomic status (SES), which may have impacted our results. Prior literature, however, has not found an association between ACEs and SES, though some studies do note that ACEs in those living in poverty may have a more pronounced impact on health [[46], [47], [48]]. Furthermore, while information on additional social determinants of health not analyzed in the current study, such as food security and food aid, were available, these measures were added at later time points in the ABCD Study; thus, while it was not possible to consider them in our analysis, we recommend future studies further explore any potential associations. Additionally, for several ACEs, including physical abuse and emotional neglect, our analyses reported inverse associations, though these values were statistically insignificant. We recommend future studies continue to explore these relationships to validate our findings and/or identify underlying mechanisms. Finally, we would like to note that while cumulative ACE scores and ACE subtypes were not associated with meeting the threshold for hypertension in young adolescents in our study, even small increases in blood pressure within the normal range have been shown to be associated with increased cardiovascular disease over time [49].

## 5 Conclusion

This study adds to the literature as it identifies preadolescence as a critical phase during which exposure to ACEs has a significant influence on blood pressure and thus subsequent cardiovascular risk. Therefore, childhood and adolescence may present an opportunity for early detection of ACEs and interventions to reduce blood pressure. Future studies should further examine the pathophysiological mechanisms underlying the association between ACEs and early elevations in blood pressure, which can help identify targeted interventions to reduce blood pressure in at-risk populations. Additionally, future studies can track the chronicity of exposure to ACEs and analyze associations of chronicity with blood pressure over time.

## Funding/Support

J.M.N. was funded by the 10.13039/100000002National Institutes of Health (K08HL159350 and R01MH135492), the 10.13039/100000862Doris Duke Charitable Foundation (2022056), and the California Collaborative for Public Health Research (CPR3).

## Role of funder/sponsor

The funders had no role in the design and conduct of the study; collection, management, analysis, and interpretation of the data; preparation, review, or approval of the manuscript; and decision to submit the manuscript for publication.

## Author declaration

We wish to confirm that there are no known conflicts of interest associated with this publication and there has been no significant financial support for this work that could have influenced its outcome.

We confirm that the manuscript has been read and approved by all named authors and that there are no other persons who satisfied the criteria for authorship but are not listed. We further confirm that the order of authors listed in the manuscript has been approved by all of us.

We confirm that we have given due consideration to the protection of intellectual property associated with this work and that there are no impediments to publication, including the timing of publication, with respect to intellectual property. In so doing we confirm that we have followed the regulations of our institutions concerning intellectual property.

We understand that the Corresponding Author is the sole contact for the Editorial process (including Editorial Manager and direct communications with the office). He/she is responsible for communicating with the other authors about progress, submissions of revisions and final approval of proofs. We confirm that we have provided a current, correct email address which is accessible by the Corresponding Author.

## CRediT authorship contribution statement

**Abubakr A.A. Al-shoaibi:** Writing – review & editing, Writing – original draft, Formal analysis. **Christopher M. Lee:** Writing – review & editing, Writing – original draft. **Julia H. Raney:** Writing – review & editing. **Kyle T. Ganson:** Writing – review & editing. **Alexander Testa:** Writing – review & editing. **Erin E. Dooley:** Writing – review & editing. **Holly C. Gooding:** Writing – review & editing. **Kelley Pettee Gabriel:** Writing – review & editing. **Fiona C. Baker:** Writing – review & editing, Methodology, Data curation, Conceptualization. **Jason M. Nagata:** Writing – review & editing, Writing – original draft, Formal analysis, Conceptualization.

## Declaration of competing interest

The authors declare that they have no known competing financial interests or personal relationships that could have appeared to influence the work reported in this paper.

## Appendix Supplementary materials

Image, application 1
