# Supplementary material for: Associations of adverse childhood experiences with blood pressure among early adolescents in the United States
Source: Am J Prev Cardiol. 2024 Oct 4;20:100883. doi: 10.1016/j.ajpc.2024.100883 (PMC11539657; doi:10.1016/j.ajpc.2024.100883)
Supplement: Supplementary file 1 [file mmc1.docx]

| Supplementary table 1. Comparison of characteristics between included and excluded participants, ABCD study at baseline. | | | |
| --- | --- | --- | --- |
| Sociodemographic characteristics | Included  (n= 4,071) | Excluded (n= 7,805) | p |
| Age | 9.8 (0.63) | 9.9 (0.62) | **<0.001** |
| Sex (%) |  |  |  |
| Female | 48.0% | 47.7% | 0.788 |
| Male | 52.0% | 52.3% |  |
| Race/ethnicity (%) |  |  |  |
| White | 54.9% | 50.4% | **<0.001** |
| Latino / Hispanic | 14.7% | 18.3% |  |
| Black | 18.6% | 20.9% |  |
| Asian | 6.2% | 5.8% |  |
| Native American | 4.2% | 3.1% |  |
| Other | 1.3% | 1.5% |  |
| Household income (%) |  |  | **0.026** |
| <$75,000 | 42.1% | 44.3% |  |
| ≥$75,000 | 57.9% | 55.7% |  |
| Parent's highest education |  |  |  |
| College education or more | 85.9% | 81.1% | **<0.001** |
| High school education or less | 14.1% | 18.9% |  |
| Number of ACEs |  |  |  |
| 0 | 21.5% | 24.2% | **0.004** |
| 1 | 32.1% | 31.5% |  |
| 2 | 22.0% | 19.7% |  |
| 3 | 11.4% | 11.2% |  |
| 4+ | 13.0% | 13.4% |  |
| ACEs type |  |  |  |
| Physical abuse (%) | 0.7% | 0.8% | 0.578 |
| Sexual abuse (%) | 0.7% | 0.9% | 0.202 |
| Emotional neglect (%) | 1.2% | 1.1% | 0.531 |
| Physical neglect (%) | 5.1% | 4.8% | 0.463 |
| Household substance use (%) | 43.7% | 39.7% | **<0.001** |
| Household divorce or separation (%) | 11.9% | 12.7% | 0.178 |
| Household mental illness (%) | 31.8% | 29.8% | **0.021** |
| Household violence (%) | 42.7% | 42.4% | 0.768 |
| Household criminal justice involvement (%) | 12.4% | 13.3% | 0.175 |
| BMI percentile, mean (SD) | 61.3 (30.9) | 59.9(30.8) | **0.024** |

Abbreviations: ACEs, adverse childhood experiences; BMI, body mass index

Appendix A. Adverse Childhood Experience (ACE) measures in the ABCD study

| ACE | File name | Question | Question | ABCD Assessment* |
| --- | --- | --- | --- | --- |
| Physical abuse | ptsd01 | ksads_ptsd_raw_762 | Shot, stabbed, or beaten brutally by a grown up in the home | KSADS-5 PTSD Module – Parent report |
|  | ptsd01 | ksads_ptsd_raw_763 | Beaten to the point of having bruises by a grown up in the home | KSADS-5 PTSD Module – Parent report |
| Sexual abuse | ptsd01 | ksads_ptsd_raw_767 | A grown up in the home touched your child in their privates, had your child touch their privates, or did other sexual things to your child | KSADS-5 PTSD Module – Parent Report |
|  | ptsd01 | ksads_ptsd_raw_768 | An adult outside your family touched your child in their privates, had your child touch their privates or did other sexual things to your child | KSADS-5 PTSD Module – Parent report |
| Household violence | ptsd01 | ksads_ptsd_raw_766 | Witness the grownups in the home push, shove or hit one another | KSADS-5 PTSD Module – Parent report |
|  | fes02 | fam_enviro6 | Family members sometimes hit each other | Environment Scale- Parent report |
|  | fes02 | fam_enviro3 | Family members sometimes get so angry they throw things | Environment Scale- Parent report |
|  | abcd_fes01 | fes_youth_q6 | Family members sometimes hit each other | Family Environment Scale – Youth report |
|  | abcd_fes01 | fes_youth_q3 | Family members sometimes get so angry they throw things | Family Environment Scale – Youth report |
| Substance abuse in the household | fhxp102 | famhx_4_p | Has any blood relative of your child ever had any problems due to alcohol such as: marital separation or divorce, laid off or fired from work, arrests or DUIs; alcohol harmed their health; in an alcohol treatment program; suspended or expelled from school 2 or more times; isolated self from family, caused arguments or were drunk a lot?** | Family History Assessment – Parent report |
| Household mental illness | fhxp201 | fam_history_13_yes_no | Has ANY blood relative of your child ever attempted or committed suicide?** | Demographics survey – Parent report  (ABCD Family History Assessment Part 2) |
|  | fhxp102 | fam_history_6_yes_no | Has ANY blood relative of your child ever suffered from depression, that is, have they felt so low for a period of at least two weeks that they hardly ate or slept or couldn't work or do whatever they usually do?** | Demographics survey – Parent report  (ABCD Family History Assessment Part 1) |
| Divorce/separation | pdem02 | demo_prnt_marital_v2 | Divorced/separated | ABCD Parent Demographics Survey |
| Criminal household member | fhxp201 | fam_history_9_yes_no | Has ANY blood relative of your child been the kind of person who never holds a job for long, or gets into fights, or gets into trouble with the police from time to time, or had any trouble with the law as a child or an adult? | Family History Assessment – Parent report |
| Emotional neglect | crpbi01 | crpbi_parent4_y | Believes in showing his/her love for me*** | CRPBI Acceptance Subscale –Youth report |
| Physical neglect | pmq01 | parent_monitor_q1_y | How often do your parents/guardians know where you are?**** | Parental Monitoring Survey–Youth report |
|  | pmq01 | parent_monitor_q3_y | If you are at home when your parents or guardians are not, how often do you know how to get in touch with them?**** | Parental Monitoring Survey–Youth report |

*All ACEs data were determined through parent and adolescent responses in the baseline assessment (2016-2018). A yes response to any of ACEs questions was counted as one-point.

**one-point given if blood relative was mother or father

***one-point if not like him/her for primary caregiver

**** one-point if never/almost never

Supplementary table 2. Associations between baseline adverse childhood experiences and hypertension at year 2 follow-up in the Adolescent Brain Cognitive Development Study.

|  | Adjusted | |
| --- | --- | --- |
|  | OR (95% CI) | p |
| Number of ACEs |  | |
| 0 | Ref |  |
| 1 | 1.01 (0.63, 1.59) | 0.982 |
| 2 | 0.77 (0.50, 1.18) | 0.215 |
| 3 | 0.90 (0.59, 1.38) | 0.612 |
| 4+ | 1.21 (0.75, 1.96) | 0.406 |
| ACE subtypes^b^ |  |  |
| Physical abuse* | - | - |
| Sexual abuse | 2.31 (0.42, 12.65) | 0.317 |
| Emotional neglect* | - | - |
| Physical neglect | 1.30 (-3.76, 6.35) | 0.599 |
| Household substance use | 0.79 (0.50, 1.27) | 0.323 |
| Household divorce or separation | 1.20 (0.79, 1.83) | 0.360 |
| Household mental illness | 1.16 (0.88, 1.53) | 0.278 |
| Household violence | 1.25 (0.91, 1.70) | 0.145 |
| Household criminal justice involvement | 0.95 (0.53, 1.68) | 0.851 |

Abbreviations: ACEs, adverse childhood experiences

*None of the participants with a history of sexual abuse or emotional neglect had hypertension

Bold indicates p<0.05

^a^Covariates: age, race/ethnicity, sex, household income, parent education, study site, and BMI.

^b^ Outputs represent the abbreviated output for a series of linear regression models with each ACE subtype as the independent variable and hypertension as the dependent variable. Thus, the table represents the output from nine regression models in total.

Supplementary table 3. Associations between baseline adverse childhood experiences and systolic and diastolic blood pressure percentiles at two-year follow-up in the Adolescent Brain Cognitive Development Study.

| Blood pressure | Systolic Blood Pressure (SBP) | | Diastolic Blood Pressure (DBP) | |
| --- | --- | --- | --- | --- |
|  | Adjusted | | Adjusted | |
|  | B (95% CI) | p | B (95% CI) | p |
| Number of ACEs |  | |  | |
| 0 | Ref |  | Ref |  |
| 1 | -0.51 (-0.05, 3.3) | 0.768 | 0.75 (-1.55, 3.06) | 0.505 |
| 2 | 1.47 (-1.24, 4.18) | 0.271 | 1.48 (-0.41, 3.37) | 0.117 |
| 3 | 0.46 (-2.59, 3.51) | 0.757 | 1.37 (-1.11, 3.84) | 0.264 |
| 4+ | 3.30 (-0.05, 6.66) | 0.053 | 2.22 (-0.64, 5.09) | 0.121 |
| ACE subtypes^b^ |  |  |  |  |
| Physical abuse | - 2.39 (-9.07, 4.29) | 0.465 | -3.39 (-16.8, 10.05) | 0.605 |
| Sexual abuse | 7.20 (-2.27, 16.69) | 0.129 | 1.72 (-3.45, 6.89) | 0.497 |
| Emotional neglect | -5.94(-13.3,1.46) | 0.11 | -1.57 (-9.31,6.15) | 0.676 |
| Physical neglect | 0.38 (-4.66, 5.43) | 0.876 | 2.88 (-0.92, 6.69) | 0.13 |
| Household substance use | **2.40 (0.41, 4.40)** | **0.021** | 1.48 (-0.31, 3.27) | 0.101 |
| Household divorce or separation | 2.03 (-0.25, 4.32) | 0.078 | 0.95 (-1.47, 3.37) | 0.422 |
| Household mental illness | 1.46 (-0.11, 3.03) | 0.067 | **2.52(1.26, 3.77)** | **<0.001** |
| Household violence | -0.72 (-3.01, 1.57) | 0.522 | -0.38 (-2.09, 1.33) | 0.653 |
| Household criminal justice involvement | -0.53 (-3.54, 2.49) | 0.72 | -2.55 (-5.97, 0.88) | 0.137 |

Bold indicates p<0.05

Abbreviations: ACEs, adverse childhood experience; SBP, systolic blood pressure; DBP, diastolic blood pressure.

^a^Covariates: age, race/ethnicity, sex, household income at baseline, household income at year 1, household income at year 2, parent education at baseline, parent education at year 1, parent education year 2, study site, and BMI.

^b^ Outputs represent the abbreviated output for a series of linear regression models with each ACE subtype as the independent variable and systolic and diastolic blood pressure as the dependent variables. Thus, the table represents the output from nine regression models in total.
